# Thriving from Work Questionnaire: Dimensionality, reliability, and validity of the long and short form questionnaires

**DOI:** 10.1002/ajim.23465

**Published:** 2023-02-07

**Authors:** Susan E. Peters, Daniel A. Gundersen, Jeffrey N. Katz, Glorian Sorensen, Gregory R. Wagner

**Affiliations:** 1 Department of Social and Behavioral Sciences, Harvard T.H. Chan School of Public Health, Boston, Massachusetts, USA; 2 Survey and Qualitative Methods Core, Division of Population Sciences, Dana-Farber Cancer Institute, Boston, Massachusetts, USA; 3 Departments of Orthopedic Surgery and Medicine, Brigham and Women’s Hospital, Boston, Massachusetts, USA; 4 Department of Epidemiology, Harvard T.H. Chan School of Public Health, Boston, Massachusetts, USA; 5 Dana-Farber Cancer Institute, Boston, Massachusetts, USA; 6 Department of Environmental Health, Harvard T.H. Chan School of Public Health, Boston, Massachusetts, USA

**Keywords:** flourishing, measurement, occupational health, quality of life, scale development, validation, work environment, work life balance, worker well-being, working conditions

## Abstract

**Introduction:**

Thriving from Work is defined as the state of positive mental, physical, and social functioning in which workers’ experiences of their work and working conditions enable them to thrive in their overall lives, contributing to their ability to achieve their full potential at work, at home, and in the community. The purpose of this study was to develop a psychometrically-sound questionnaire measuring the positive contribution that work can have on one’s well-being both at, and outside of, their work.

**Methods:**

We used both a qualitative and quantitative approach of item reduction, domain mapping dimensionality testing, development of “long-” and “short-” versions of the questionnaire, reliability, and construct and criterion validity testing. This was established in two independent online samples of US based workers (*n* = 1550, *n* = 500).

**Results:**

We developed a bi-factor model 30-item long-form and a uni-factorial 8-item short-version. The long-form measures both the latent construct of Thriving from Work and six domains (psychological/emotional; work-life integration; social; experience of work; basic needs; health). Both long- and short- forms were found to have high empirical reliability (0.93 and 0.87 respectively). The short-form captures 94% of variance of the long-form. Construct and criterion validity were supported. Test-retest reliability was high.

**Conclusions:**

The Thriving from Work Questionnaire appears to be a valid and reliable measure of work-related well-being in United States workers. Further testing is needed to refine and test the instrument in specific industries, unique worker populations, and across geographic regions.

## 1 | INTRODUCTION

Work is an integral part of the health and well-being of working-age people, their families, and communities.^1^ The average worker spends about 90,000 h at work over their lifetime, equating to about a third of their waking hours each week.^2^ Being employed—and the quality of the conditions of work that one is exposed to—is an important determinant of health and well-being.^3^ Engaging in productive work plays a central role in people’s lives, providing a sense of meaning, purpose, and social identity, as well as being necessary for both economic and social development in society.

There is increasing support for broader conceptualization of work-related well-being that focuses on how work can improve the quality of people’s lives,^4–6^ and a call for instruments to move away from narrow conceptualizations of worker well-being (such as job satisfaction or employee engagement) towards those that apply a more comprehensive view of worker well-being that considers multiple dimensions.^5^ There is no consensus around a single definition of subjective well-being, or of work-related well-being.^7,8^ However, thriving is generally considered to be the highest level of well-being obtainable, a consequence of the interplay between both individual traits and external environmental exposures.^4^ Workers’ experiences of their jobs, working conditions, and the work they perform can contribute to whether, or not, they thrive both at work and in their lives outside of work.

Thriving workers are likely to have cascading benefits for organizations: workers with higher levels of well-being incur lower healthcare costs, are more productive, and are more likely to remain with that employer for longer periods.^9^ Identifying the extent to which workers are thriving could also facilitate employers’ implementation of policies, programs, and practices designed to support worker well-being and create thriving workplaces and workforces. Considering well-being as an important worker outcome in the workplace may also be a powerful pathway for addressing equity,^10^ and contribute to building a culture of health for both workers^8^ and the organizations in which they work.^11^ This new instrument advances the measurement of work-related well-being by capturing the interconnected nature of work and its impact on well-being both at work and outside of work.

Due to the lack of a definition of thriving as it relates to one’s work, in a previous study, we conducted a multistage iterative process to conceptualize this concept—“Thriving from Work.”^12^ We defined Thriving from Work (TfW), an integrated subjective measure of work-related well-being, as “the state of positive mental, physical, and social functioning in which workers’ experiences of their work and working conditions enable them to thrive in their overall lives, contributing to their ability to achieve their full potential in work, home, and community.”^12^ We conceptualized TfW across seven dimensions including psychological, emotional, social, work-life integration, basic needs for thriving from work, job design and experience of work, and health and physical and mental well-being from work. We mapped 37 attributes to these dimensions and cognitively tested candidate items to each of these attributes. We identified 87 possible items that had good face validity and cognitive response properties for measuring the attributes of TFW.^12^ In this current study, we build on this previous work to describe the development and validation of a new questionnaire for the comprehensive assessment of workers’ TfW–Thriving from Work Questionnaire (TfWQ). Based on our definition and conceptualization of TfW, the purpose of this instrument is to measure the extent to which workers are thriving from their work, acknowledging that work impacts our well-being holistically both at work and outside of work.

The TfWQ adds to the existing literature on well-being from work as it responds to calls for a more comprehensive measure of well-being from work that considers the interconnectedness and bi-directional influence of work, and our lives and responsibilities outside of work. Previous questionnaires have focused on narrower conceptualizations or have used proxies, such as job satisfaction, or employee engagement, to measure worker well-being or thriving. For example, the “Thriving at Work” questionnaire developed by Porath et al.^13^ focuses only on how work impacts vitality and learning as a consequence of workers’ experiences at work. Eaton et al.’s^14^ Work Well-being Index focuses on the social and psychological attitudes towards one’s job. The Employee Well-being Scale, developed by Zheng and colleagues, focuses on job performance and organizational commitment. Warr and Russell, and Daniel’s scales measure affective well-being at work.^15,16^ Orsila et al.^17^ developed the more comprehensive Work-related Well-being Questionnaire, measuring dimensions of workplace culture, physical and mental health, job satisfaction, meaning and affect; however, this questionnaire has little consideration of how workers’ experiences of their working conditions, and whether their basic needs, such as fair pay, are being met by their work and how this affects their well-being. A useful integrated assessment tool is the 58-item National Institute of Occupational Safety and Health (NIOSH) Worker Well-being Questionnaire (WellBQ) which provides an evaluation of the determinants of worker well-being across life (e.g., general health) and work dimensions,^18^ but does not provide an assessment of a latent construct of worker well-being. Further, many occupational health metrics focus on the negative impacts of work and working conditions on health and well-being. A recent review by Fox et al.^19^ found that there was much heterogeneity in both the conceptualization and measurement of worker-well-being, with the proxy of “job satisfaction” still being used in most intervention studies.

The TfWQ has several innovations that add to the existing literature on worker well-being and the advancement of measuring work-related well-being. The TfWQ focuses on the *positive* contributions that work has on workers’ thriving in the workplace and community and considers the diversity of dimensions and specific attributes that comprise work-related well-being. We developed both a long- and short-form of the TfWQ to enable broad application across research, policy, and practice including as: (a) an outcome measure of workers’ thriving for research purposes,(b) a one-time survey instrument for use across a working population or for periodic surveillance (repeated surveys) across a diversely employed worker population or within a single employing organization as policies are implemented and/or working conditions change, and (c) an organizational diagnostic tool to identify priority areas for interventions to improve worker well-being and to monitor their effectiveness across different dimensions of workers’ thriving.

The purpose of this study was to develop reliable and valid long- and short-versions of the TfWQ. Thus, in addition to presenting the development of the instrument in this paper, we respond to two foundational questions posed by the development process: Is the instrument reliable and is it valid?

## 2 | METHODS

Our development of the TfWQ was based on the Promise of PROMIS framework.^20^ Using this framework, we used a comprehensive qualitative approach,^12^ followed by a quantitative process of item reduction, domain mapping dimensionality testing, development of “long” and “short” form versions of the questionnaire, and reliability and validity testing (Table 1).

### 2.1 | Participants and data collection

This study used two worker samples generated from an online research participant recruitment service Prolific Academic Ltd. (Prolific.co). This online platform has been used successfully to recruit samples by various academic researchers,^21–25^ including the development and validation of questionnaires.^26,27^ At the time of the study (October 2020), the platform had about 267,000 participants in the United States. We requested that Prolific.co generate two independent samples of workers for two different timepoints—a development sample and a validation sample—with the following eligibility criteria: current US based worker with a full-time, part-time, or casual/hourly job. The benefit of using this online research-based platform is that workers are vetted before joining the platform as a research participant. Other online platforms, such as Amazon Turk, or using social media outlets, as a recruitment method, do not have similar vetting processes. Workers could be gig or contract workers or have more than one job.

We only included US based workers for this first development and validation study. This is because cultural differences between countries exist due to differing social, political, and economic environments, as well as employment and labor patterns that are likely to impact how organizations implement policies, programs, and practices and that these policies, programs, and practices are likely to affect worker well-being.^28^

### 2.2 | Methods to increase quality of survey responses

To increase the quality of survey responses we employed several measures. In Qualtrics XM (Qualtrics), we enabled bot detection and Google’s RecaptchaScore: both provide an indication of whether the respondent is likely to be a bot. We also enabled fraud detection measures that do not allow duplicate responses and identify participants who are likely to be answering the survey more than once (e.g., using geographic location parameters and IP address). Due to the Prolific.co process of vetting the research participants to join their panel, we were confident that the possibility of bots being used was low. However, we erred on the side of caution by including these additional security measures. We had no responses from bots or duplicate responses identified using our additional security measures, further ensuring the legitimacy of participants’ responses. To reduce rapid responder bias in our sample, we also excluded survey responses that were more than three standard deviations below the mean for completion time, or that failed one of the three attention checks contained in the survey (these were questions designed to see if the participant was paying attention by stating, “please respond ‘always’ to this question”). We piloted the electronic survey with 50 participants to ensure no programming errors (e.g., were skip patterns functioning as expected?). No programming issues were identified, and thus no changes were made to the electronic survey.

### 2.3 | Development sample: Sample used to develop the long- and short-form questionnaires

The first sample (hereafter referred to as the “development sample”) was used to develop the long- and short-versions of the questionnaire. This sample consisted of 1550 workers (an additional 85 respondents did not proceed past the consent page). This sample was used to identify the items comprising the final long- and short-versions of the TfWQ via multidimensional item response theory (IRT) analysis. We also used this sample to assess scale reliability and concurrent validity of both TfWQ versions. We fielded the development sample survey in October 2020.

### 2.4 | Validation sample: Sample used to validate the questionnaires

The second sample (referred to as the “validation” sample’) was used to validate the questionnaire that we developed. This sample consisted of 500 workers (an additional 12 respondents did not proceed past the consent page). We, first, fit a confirmatory model of the items identified in the first sample, and then, we assessed test–retest reliability. We fielded the validation sample survey in May 2021. We then disseminated the survey again to a random sample of 100 of the validation sample respondents in June 2021 to establish test–retest reliability.

### 2.5 | Survey instrument

#### 2.5.1 | Survey content

The development survey instrument included candidate TfWQ items developed through formative research activities (Phase 1, Table 1) and are described fully in Peters et al.^12^ These activities included a literature review, workshop, review of existing questionnaires to identify relevant items and development of new items, interviews with international experts and review of versions of the questionnaire through an iterative process, and four rounds of cognitive testing with US based workers from different occupations and backgrounds. Items had closed-ended Likert-type response options (never, rarely, sometimes, usually, almost always, always). Items were displayed in a randomized order within their hypothesized dimensions to mitigate items displayed later in the survey being more affected by potential respondent satisfaction. To assess construct validity (an assessment of how well the TfWQ items represent the latent construct of TfW), we compared the TfWQ with a set of measures that assess similar constructs that should be related to TfW, but not so similar that they are very highly correlated. These measures were: an adapted version of Gallup’s Cantril’s Ladder that assesses whether one is thriving in life (or not),^29,30^ Life Satisfaction measured with a four-point (very satisfied to very dissatisfied) single item ordinal scale,^31^ mental health using the K-6 five-point Likert scale (“all of the time” to “none of the time” with a higher score indicating worse psychological distress,)^32^ and Best Job relative to their current job using a Cantril’s Ladder approach, similar to Gallup’s Thriving scale, developed by the research team. In the Best Job question, participants were asked to imagine that each step of the ladder is a number from 0 to 10, with 10 representing their perceived best job. Participants were asked to indicate which step on the ladder represents their current job relative to their perceived best job. The survey also included demographic (e.g., age, sex, gender, education level, ethnicity/race, annual income) and occupational questions (e.g., number of jobs, hours worked, employment status, tenure). The validation survey instrument contained an abbreviated version of the measures used in the development survey.

#### 2.5.2 | Survey methods and process

We programmed the survey in Qualtrics XM (Qualtrics). For both samples, invitations were sent out by Prolific.co as the known entity to the participants. Participants consented to participate in the study by opting to complete the survey. Surveys were received anonymously by the research team; although each participant had a unique identifier which Prolific.co could link to survey participants in a re-identifiable way, the researchers only received a copy of the completed survey through Qualtrics with the participants’ unique identifiers. Participants received conditional incentives of $6 for completing the development and validation surveys, and an additional compensation payment of $1.50 for the follow-up retest sample. The development survey took a median time to complete of 18.5 min (IQR:13.9,26.4), 9 min (IQR:7.5,13.2) to complete the validation survey, and 7.3 min (IQR:5.2,9.6) for the retest survey.

In the development sample, the survey was sent to 16,312 active Prolific survey participants who met our eligibility criteria. Due to funding constraints, we closed the survey once we reached 1550 participants who had completed the survey (an additional 85 participants opted not to complete the survey). No further participants could complete the survey after it was closed. For the validation, we requested that Prolific not include participants who completed the survey in the first round: 18,885 participants were eligible to participate, and the survey was closed once we received 500 surveys (an additional 12 participants opted not to complete the survey). For retest reliability, 100 individuals were randomly selected from the 500 validation sample participants. All 100 individuals invited completed the survey.

### 2.6 | Statistical analyses

#### 2.6.1 | Developing the long-form

Items were first screened for large item-nonresponse, as well as floor- and ceiling-effects, which we defined as items with greater than 10% nonresponse or ≥80% selecting the highest or lowest response category. These items were flagged and discussed for possible elimination by the investigator team.

**FIGURE 1:**
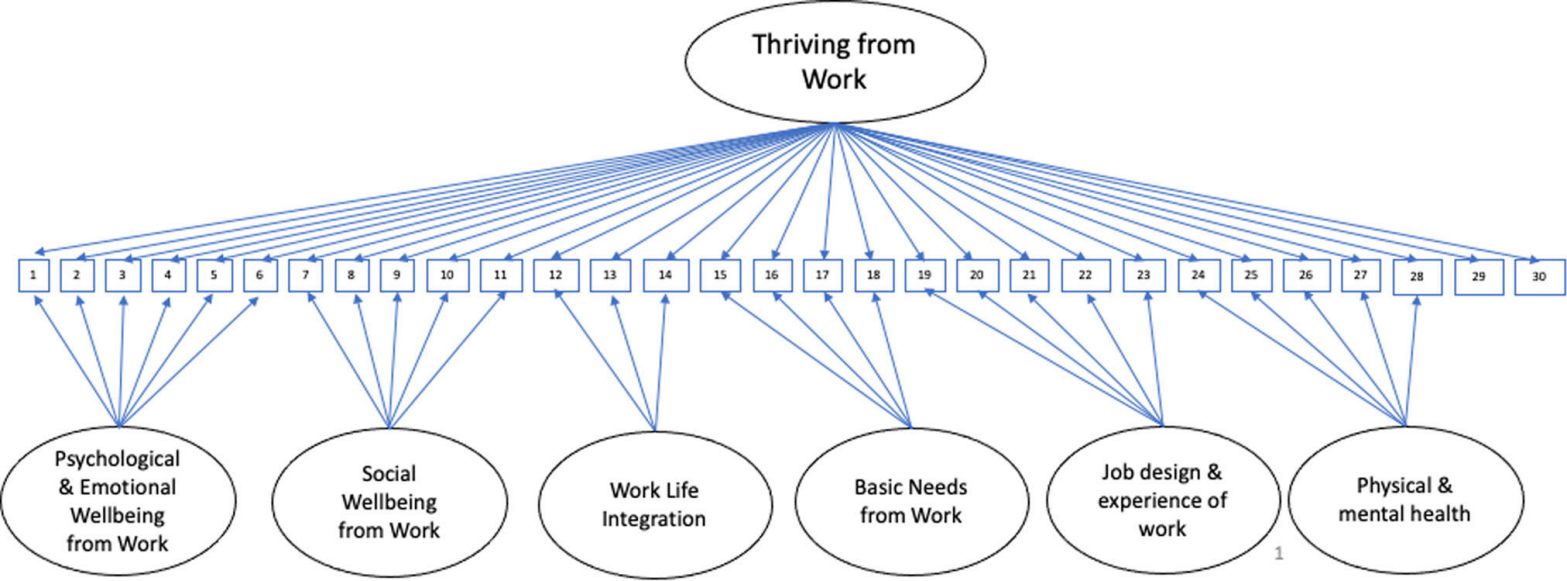
Bi-factor Model for the Long-form Thriving from Work Questionnaire displaying how the items loaded on to both Thriving from Work latent construct and the six theorized dimensions of Thriving from Work. Each small box represents at item number. [Color figure can be viewed at wileyonlinelibrary.com]

Using the development sample, we estimated a bifactor graded response IRT model (Figure 1) with 1 general and 6 specific domains using all 87 candidate items. The bifactor model was chosen because the theory of TfW, as described previously,^12^ is such that we hypothesize a general TfW domain and specific domains of TfW (e.g., social well-being from work, basic needs for thriving from work, etc.) that are uncorrelated with each other. The bifactor graded response model allows us to evaluate the relationship of each ordinal item with a general TfW latent construct and, conditional on general TfW, their hypothesized specific domain. As such, all items were specified to load on the general TfW domain and on their hypothesized specific domain. We eliminated items if (1) they were judged by the investigators to have small estimated marginal discrimination parameter on general TfW relative to the remaining items, or (2) violated the local independence assumption (i.e., had substantial residual covariance with another item after conditioning on general TfW and their domain’s specific factors). When an item-pair was found to have local dependence, the item with the largest estimated marginal discrimination parameter on general TfW was retained, unless that was judged to compromise content validity. If items had small estimated discrimination parameters on their hypothesized specific domain, the model was re-specified with those items loading only on the general TfW domain.

We judged the evidence of a general TfW domain by the ratio of the largest to second largest eigenvalues of the polychoric correlation matrix and the explained common variance (ECV) due to the general domain. Model fit was evaluated by limited information M2 statistics,^33,34^ as well as root mean squared error of approximation (RMSEA), standardized root mean squared residual (SRMSR), and the comparative fit index (CFI).^33–35^ We used common cut-points of RMSEA, or its 90% confidence interval (CI) lower bound <0.05, SRMSR < 0.08, and CFI > 0.95, as guides to judge model fit.^36^ These represent the fit of the model relative to a saturated model (M2), absolute fit (RMSEA), the model implied correlation matrix relative to observed correlation matrix (SRMSR), and relative to a null model (CFI). As these are different fit metrics, we did not judge based on strict cut-offs. Rather, we emphasized interpretation of all metrics, on balance, to judge the fit of the models.

#### 2.6.2 | Developing the short-form

To develop a short-version of the questionnaire, we selected the items from each domain that had the highest marginal discrimination parameter on the general TfW factor and judged by the investigator team to retain content validity. We then fit a unidimensional graded response model and eliminated items using the same criteria described above. The unidimensional model fit was evaluated using the M2, RMSEA, SRMSR, CFI with cut-points and interpretation as described above. Empirical reliability was calculated as a summary measure of scale reliability for general TfW (long- and short-forms) and each specific factor of the long-form. All analyses were conducted using the mirt package in R.^37^

#### 2.6.3 | Assessing validity and reliability

To validate the measurement model identified in the development sample, we fit the same models using the validation sample. We compared the consistency of discrimination parameters to the calibration sample using point estimates and 95% CIs, and we inspected model fit statistics as described above. To assess test–retest reliability, we calculated the intracluster correlation coefficients for the modal a-posteriori scores for the long- and short-forms for respondents who were in the validation and retest samples. We considered test–retest reliability to be good if ICC > 0.70, meaning 70% of the variance of the TfW scores were due to between-participant variation rather than within-participant variation from the first to second measurements.

## 3 | RESULTS

### 3.1 | Participants demographic characteristics

The development sample was mainly white (77%), non-Latino (91%), identified as male (54%), had 1 job in the past month; and the plurality had a Bachelor’s degree (44%), were 30–39 years of age (38%), had an annual family income of $70,000 to $99,999, and had a median tenure of 4.2 years at their main job (Table 2). Workers represented a broad range of sectors with the highest representation from education (15%), healthcare (11%), finance (8%), retail (7%), information technology (6%), and manufacturing (5%). The sample characteristics for the validation and re-test samples were very similar to the development sample, though the re-test sample had a higher proportion who identified as male (62%) and the validation sample a slightly lower median tenure at the main job (3.8 years).

### 3.2 | Long-form Thriving from Work Questionnaire

Using the development sample, candidate item descriptive statistics were calculated (Supporting Information: Appendix 1). No items exhibited floor or ceiling effects. The three commute items had item nonresponse greater than 10% (12.4%, 10.8%, and 10.5%). The investigator team determined that eliminating all three commuting items would compromise content validity. Because they had similar levels of nonresponse, all three were retained for initial evaluation in the bifactor IRT model.

The ratio of the first to second eigenvalues from the polychoric correlation matrix for all candidate items was 5.11 and 7.29 for the bifactor model of the long-form TfWQ. Of the 73 candidate items we included for consideration, 43 were eliminated due to the low marginal discrimination parameters and psychometric performance or because we did not consider them to add to the clinimetric profile of the TfWQ. Table 3 presents the estimated marginal discrimination parameters for each item on general TfW and six specific domains of the final long-form TfWQ, as well as the ECV and empirical reliability for each domain, and overall model fit statistics. The ECV for general TfW was 5.7%, and 71%, 9.8%, 3.3%, 4%, 3%, 3% for the six TfW domains, Psychological and Emotional Well-being from Work, Social Well-being from Work, Work/life integration, Basic Needs for TfW, Experience of Work and Job Design, and Health and Physical and Mental Well-being from Work, respectively. The marginal discrimination parameters for general TfW factor ranged from a low of 0.66 (“Injury” item) to a high of 2.92 (“Respect” item), with a median of 1.7 across all items. Across the six domains, Social Well-being had the greatest discrimination parameters (all >2). while Physical and Mental Well-being had the smallest discrimination parameters and were more variable (range 0.66–1.92). For the specific domains, the marginal discrimination parameters ranged from 1.01 to 1.42 for Psychological and Emotional Well-being, 0.31–1.03 for Social Well-being, 0.50–1.44 for Work-Life Integration, 0.53–1.02 for Basic Needs, 0.35–1.06 for Experience of Work and Job Design, and 0.21–1.67 for Physical and Mental Well-being. The “Stress from Work” item had negative marginal discrimination parameters for the general TfW and Physical and Mental Well-being domains. Model fit was M2_(*df* = 257)_ = 854.9 (*p* < 0.001), RMSEA = 0.05 (RMSEA 95% CI lower bound [90% CI-LB] = 0.04), SRMSR = 0.05, and CFI = 0.96. The empirical reliability was 0.93 for general TfW, 0.82 for Psychological and Emotional Well-being, 0.62 for Social Well-being, 0.70 for Work-Life Integration, 0.52 for Basic Needs, 0.54 for Experience of Work and Job Design, and 0.62 for Physical and Mental Well-being. Two items (Voice and Recognition) only loaded onto general TfW, and did not load on to their originally hypothesized domain. Figure 1 presents the final bifactor model with the items loading on to the general TfW Factor and the six domains.

### 3.3 | Short-form Thriving from Work Questionnaire

Using the development sample, we then estimated discrimination parameters for the 8-item short-form TfWQ. These ranged from 1.73 (Emotional Well-being: Enthusiasm) to 2.54 (Social Well-being: Fairness (1). These were similar to the marginal discrimination parameters on the general domain of the long-form TfWQ (Table 4). Model fit statistics were M2_(*df* = 20)_ = 253.81 (*p* < 0.001), RMSEA = 0.09 (90% CI-LB = 0.08), SRMSR = 0.05, and CFI = 0.97. The empirical reliability of the short-form was 0.87.

### 3.4 | Establishing validity and retest reliability

**FIGURE 2:**
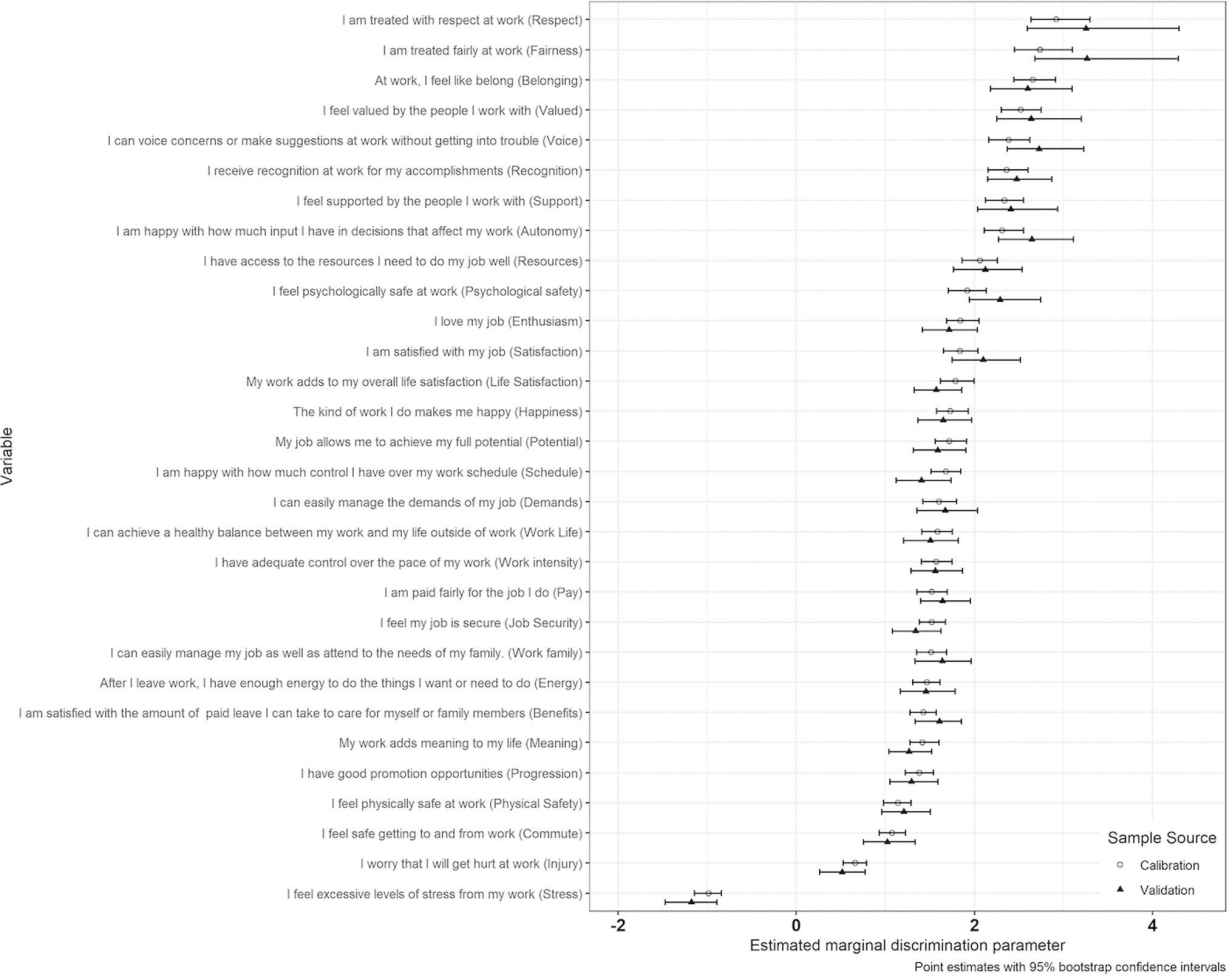
General Thriving from Work Consistency for the long-form: calibration and validation samples.

**FIGURE 3:**
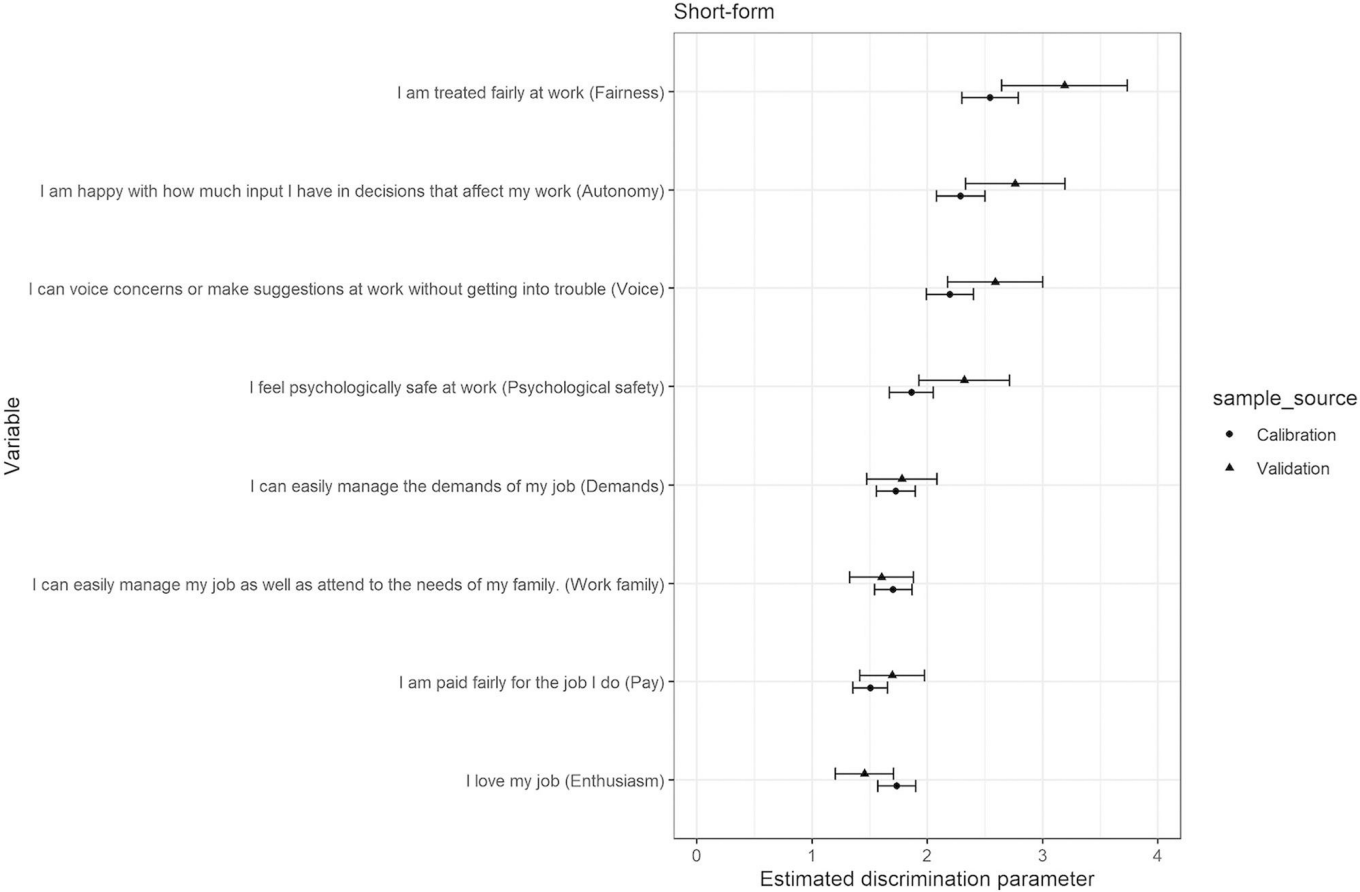
General Thriving from Work Consistency for the short-form: calibration and validation samples.

We then used the validation sample to examine both the long- and short-form models’ fit. We found that the validation models produced consistent marginal discrimination parameters when compared to the development sample (Figures 2 and 3), with each item having overlapping 95% CI. The intracluster correlation coefficient for the validation sample and retest sample was 0.89 (95% CI: 0.84–0.92) and 0.84 (95% CI: 0.77–0.89) for the long- and short-forms, respectively.

To establish construct validity, the long-form is positively correlated with Cantril’s Thriving Ladder (*r* = 0.37), job-related Cantril’s Ladder (*r* = 0.48), general life satisfaction (*r* = 0.43), and negatively correlated with overall mental health (*r* = −0.45) (Table 5). The TfWQ has a negative correlation with mental health as a higher mental health score indicates poorer mental health status. Likewise, the short-form is positively correlated with Cantril’s Thriving Ladder (*r* = 0.36), job-related Cantril’s Ladder (*r* = 0.48), general life satisfaction (*r* = 0.42) and negatively correlated with overall mental health (*r* = −0.45).

The long- and short-form TfWQ are strongly positively correlated (*r* = 0.97). The final reproducible, reliable, and valid versions of the TfWQ can be found in Supporting Information: Appendix 2.

## 4 | DISCUSSION

Historically, occupational health research has focused on identifying work hazards resulting in injury or illness and the interventions for controlling risk. Current scientific investigations of the relationships between work and work exposures to worker health are evolving to incorporate an expanded focus on positive human functioning to understand what makes people thrive and flourish both at and beyond work.^5^ The concept of “Thriving from Work” responds to the call for an expanded view of occupational safety and health by measuring positive work-related well-being across several dimensions.^38^ We developed and conducted preliminary psychometric evaluation of the long- and short-form versions of the TfWQ in the United States. We responded to our research questions by establishing that both the long- and short-form versions of the TfWQ are reliable and valid measures of worker well-being. Our current testing in a US sample of workers from different industries and backgrounds found both the long- and short-form versions of the questionnaire to be reproducible, reliable, and valid—important foundational properties of a newly developed questionnaire. These properties allow us to move on to the next stages of psychometric testing—in different worker populations, across different sectors, and in different languages/settings.

Interest in the different types of well-being and the measurement of well-being have increased greatly over the last several years. The World Health Organization’s definition of health places well-being as a central tenet,^39^ and the US Surgeon General recently highlighted the important role that work has on worker well-being, providing an important setting for population health well-being interventions.^1^ In the occupational health field, worker self-administered questionnaires are increasingly being used to evaluate perceptions of workers’ experiences of, and their satisfaction with, their work, working conditions, and also their health and well-being. However, many of these instruments have known limitations, and none, to our knowledge, focuses on workers’ thriving holistically considering both the workplace and in their lives outside of work.

The COVID-19 pandemic brought unprecedented attention to work as a key factor in the health status and well-being of workers, their families, and the communities in which they live as well as the social and economic vitality of their countries.^40^ A survey of working age US adults found that these challenges brought forth by the COVID-19 pandemic impacted people’s general well-being in significant ways.^41^ Additionally, a 2020 Gallup survey found that in 2020, less than 50% of working age adults were “thriving” in life (as determined by Cantril’s life satisfaction scale), with the majority of adults “suffering” or “struggling.”^42^ The effects of the pandemic reflected the diverse, complex, broad, and interconnected forces that impact both worker and population well-being. The pandemic has accelerated changes in labor markets, adoption of technologies, and, to some extent, attitudes towards work both for those who must work at locations outside their homes and for those able to work remotely.^40^ Employers are redesigning jobs and policies and practices to support new and emerging conditions of work and job demands.^40^ These changes in working conditions will inevitably affect the extent to which workers thrive while working and outside of work, and have emphasized the important need for us to study and measure work-related well-being.

### 4.1 | Applications for the Thriving from Work Questionnaire

Spurred by the lack of an instrument designed specifically to measure thriving from work, we developed and tested three versions of a questionnaire that could be used for different purposes. First, we conceptualized TfW across six domains and mapped attributes of TfW to these domains using an iterative comprehensive questionnaire design process. These items—as detailed in a previous publication—could be used as a diagnostic tool to identify attributes in the workplace that require attention to improve worker well-being.^12^

From the original set of items, we then developed a long-form questionnaire with the goal of measuring the latent construct of TfW, a measure of work-related well-being, as well as our conceptualized domains of TfW. The long-form can be used as an outcome measure or to provide well-being data on the extent to which workers or populations of workers are thriving from their work. The long-form has a bifactor model allowing us to measure the latent construct of TfW, but also considers that TfW has several dimensions: Psychological and Emotional Well-being from Work; Social Well-being from Work; Work-Life Integration; Experience of Work and Job Design; Basic Needs for TfW; and Health and Physical and Mental Well-being from Work.

The psychometric testing we have conducted to date indicates that the long- and short-form versions are reproducible and valid in a US worker population. These are foundational psychometric properties that enable us to move onto the next steps of instrument development. Future studies can examine more subtle issues such as subscale structure of the dimensions of TfW and improving the ability of these dimensions to measure their latent construct (e.g., social well-being from work), different methods/models for scoring, and group measurement (e.g., can we measure thriving work groups or workforces?) and translating and adapting the questionnaire for different geographical and cultural settings. Of note, we had originally considered the Psychological and Emotional Well-being domains (as described in Peters et al.^12^ as being theoretically distinct. However, our analysis found that items from these two domains loaded onto the one factor and were conceptually considered the same by participants. Thus, they were combined into one domain of TfW. Similarly, some of the subdomains of the questionnaire did not have strong enough empirical reliability for us to recommend using these as stand-alone instruments. One domain, psychological and emotional well-being had good reliability (0.82); this domain could be used as a stand-alone measure of this construct. Thus, additional research is needed to further refine the other domains if they are to be used as a domain subscale.

Lastly, we developed a short-form, that, like the long-form, is a measure of the latent construct TfW, and has utility in worker surveys where survey length needs to be considered. The short-form questionnaire is a reliable and valid measure of the latent construct of TfW capturing each dimension of the long-from. We also tested the short-form, swapping out the Psychological Safety item for the Physical Safety item, with no change in the model characteristics or robustness of the questionnaire’s reliability. This has important implications for the utility of the short-form as certain populations of workers may have different experiences of physical safety or psychological safety at work due to job demands, characteristics of the workplace, or the reason for measuring TfW in the worker population. For example, psychological safety may be more relevant for office-based jobs, whereas physical safety may be more of a concern in manual laboring occupations. In future iterations of the questionnaires, we aim to test a general safety question to examine if it performs similarly to both the physical and psychological safety questions. At this stage, we recommend selecting the item (either Physical Safety or Psychological Safety) that is most relevant for the worker population when using the short-form.

### 4.2 | Policy implications

The measurement of well-being is essential for understanding worker and population health. Measurement of well-being at individual and population levels enables governments, policy makers, employers, and worker advocates to identify how work is benefiting or adversely affecting quality of life at one point in time or over a period of time. A granular evaluation of the influence of specific working conditions will enable the possibility of targeting interventions intended to improve workers’ thriving and evaluating the consequences of their implementation for workers, their families, and communities, as well as the employing enterprises. Accurate measurement and ongoing monitoring can identify priority areas for improvements in both public and enterprise-specific policies and assist in the evaluation of the success of such interventions. Further, it allows comparisons to be made across, for example, worksites, employers, communities, regions, and countries. Well-being data can inform priority-setting, drive change in areas of need or identify best practices in places where people are thriving.

The implementation of policies, programs, and practices to improve the quality of work and work environments are of social and economic benefit for individuals, their employers, and broader society. A recent systematic review examining the effectiveness of organizational interventions to improve dimensions of worker well-being found that improvements in working conditions resulted in higher levels of general well-being, work-specific well-being, and work-family well-being.^19^ Workforces with high reported well-being have also been found to be healthier, more engaged at work, more productive and less likely to leave their job.^6,43,44^

### 4.3 | Study strengths and limitations

The samples used in this study, while being representative of an online worker sample, may not be representative of the general US worker population. Using an online sample is a time-efficient method for conducting preliminary psychometric evaluation of the novel questionnaires and has been used in the development of other questionnaires. A strength of using an online survey through a research crowdsourcing platform, such as Prolific.co, is that the samples are more likely to be demographically diverse than limited laboratory samples. These platforms can be prone to rapid responder bias; however, we attempted to overcome this by not including worker survey responses in the final data sets that were below three standard deviations of the mean completion time. Another risk of using an online survey is that respondents may not legitimately meet the eligibility criteria, answer honestly, or may even employ bots to respond. This was a primary reason why we selected Prolific.co, as they vet their research participants and have implemented methods to overcome these limitations. In addition, we also implemented quality checks (as described in the Section 2) and found that our sample had no bots, or double respondents. Other questionnaires are often initially tested on students or in specific worker industry samples; whereas we were able to obtain a fairly diverse sample of workers. While the instrument was developed in the United States and potentially may not be representative of worker samples in other countries, we are currently conducting further studies to validate the instruments in other countries, and in different languages. In addition, further validation studies are needed to capture those with different work experiences, across different occupations and sectors, across different wage levels and positions, and across demographically and occupationally diverse worker groups.

Much of the questionnaire development was conducted in a large part during the COVID-19 pandemic. In person research was suspended during this time, so using an online research platform to complete this study was necessary and effective. The validation of the questionnaire during the pandemic may not have been an ideal, but many of the changes brought about by the pandemic are expected to endure.^40^ For example, increases in the gig workforce and workers in nonstandard work arrangements globally were evident before the pandemic—the challenges they face were merely emphasized by the pandemic impact. In high- and middle-income countries, many workplaces have transitioned to decentralized and remote locations linked by technology. This is likely to continue, with up to 25% of workers globally expected to remain in hybrid work arrangements.^45^

The present analysis found good empirical and test–retest reliability as well as validity of our new tool, which was initially evaluated in a diverse national sample of workers from different industries and of different backgrounds, and then confirmed in a new sample of workers from the same population. Further testing of the tool could focus on populations outside the United States, unique worker populations such as workers in specific sectors, and translations of the instrument.

## 5 | CONCLUSIONS

The TfWQ appears to be a robust measure of work-related well-being which has been designed to have broad utility across research and practice, facilitating the design, implementation, and evaluation of changes in policies and practices influencing the conditions of work. The TfWQ can be used to identify priority areas for intervention and, through use over time, may clarify the changes that benefit the well-being, satisfaction, and functioning of workers as well as social and economic outcomes of interest to employers.

## Supplementary Material

Appendix 1

Appendix 2

SUPPORTING INFORMATION

Additional supporting information can be found online in the Supporting Information section at the end of this article.

## Figures and Tables

**TABLE 1:** Phases of development for the Thriving from Work Questionnaire.

Phase	Activities
Phase 1: Conceptualizing thriving from work, its dimensions and identification, development and qualitative testing of candidate items	• Review of international scientific literature on worker well-being, thriving and existing measurement instruments • Workshop with 33 researchers from diverse disciplines to obtain input on conceptualizing thriving from work, its dimensions, and attributes • Review of existing questionnaires to identify relevant items and development of new items where no existing items could be found • Interviews with 18 international content experts to obtain input on the dimension, attributes, and items for the TfWQ • Four rounds of cognitive testing interviews with 26 workers from diverse occupations and backgrounds • Review by the same 18 international content experts for final feedback • Eighty-seven candidate items mapped to 37 attributes across 7 dimensions were identified
Phase 2: Item reduction to develop long-form and short form questionnaires	• Online survey using the Prolific.co research participant platform of *n* = 1550 workers • Long form questionnaire was developed consisting of 30 items with 6 dimensions • Short form questionnaire was developed consisting of eight items
Phase 3: Reliability and validity testing	• Conducted reliability and construct and criterion validity analyses
Phase 4: Confirmatory testing and re-test reliability	• Conducted a second sample with 500 different workers using Prolific.co • Random sample of 100 of these workers completed a second survey 1 month later • Conduct re-test reliability and confirmatory analyses

*Note:* Phase 1 is reported in a previous publication.^7^ This paper describes Phases 2–4.

Abbreviation: TfWQ, Thriving from Work Questionnaire.

**TABLE 2:** Description of the US worker samples.

Characteristic	Calibration *N* = 1479^a^	Validation *N* = 509^a^	Retest *N* = 101^a^
Race			
White	1134 (77%)	394 (77%)	80 (79%)
Black/African American	107 (7.2%)	29 (5.7%)	11 (11%)
Asian	146 (9.9%)	58 (11%)	7 (6.9%)
Native American, Alaskan Native	13 (0.9%)	3 (0.6%)	1 (1.0%)
Other	79 (5.3%)	25 (4.9%)	2 (2.0%)
Ethnicity			
Hispanic or Latino	130 (8.8%)	30 (5.9%)	8 (7.9%)
Not Hispanic or Latino	1,349 (91%)	477 (94%)	93 (92%)
Unknown		2	
Gender			
Female	655 (44%)	225 (44%)	37 (37%)
Male	806 (54%)	275 (54%)	63 (62%)
Transgender	8 (0.5%)	3 (0.6%)	0 (0%)
Do not identify as male, female, or transgender	10 (0.7%)	5 (1.0%)	1 (1.0%)
Unknown		1	
Highest level of education			
<HS	3 (0.2%)	2 (0.4%)	1 (1.0%)
HS/GED	104 (7.0%)	35 (6.9%)	10 (9.9%)
Some college	205 (14%)	74 (15%)	18 (18%)
Associates degree	118 (8.0%)	33 (6.5%)	6 (5.9%)
Postsecondary certificate	23 (1.6%)	5 (1.0%)	0 (0%)
Bachelor's degree	658 (44%)	234 (46%)	48 (48%)
Graduate degree	368 (25%)	126 (25%)	18 (18%)
Age			
19 or younger	26 (1.8%)	10 (2.0%)	1 (1.0%)
20–29	522 (35%)	178 (35%)	28 (28%)
30–39	562 (38%)	212 (42%)	47 (47%)
40–49	207 (14%)	64 (13%)	13 (13%)
50–59	105 (7.1%)	33 (6.5%)	7 (6.9%)
60–69	52 (3.5%)	9 (1.8%)	3 (3.0%)
70 or older	5 (0.3%)	2 (0.4%)	2 (2.0%)
Unknown		1	
Number of jobs in last month			
1	1248 (84%)	432 (85%)	84 (83%)
2	185 (13%)	59 (12%)	14 (14%)
3	35 (2.4%)	14 (2.8%)	3 (3.0%)
4 or more	11 (0.7%)	2 (0.4%)	
Unknown		2	
Annual family USD Income			
Less than $25,000	94 (6.4%)	38 (7.6%)	11 (11%)
$25,000–$49,999	299 (20%)	88 (18%)	21 (21%)
$50,000–$69,999	279 (19%)	92 (18%)	24 (24%)
$70,000–$99,999	326 (22%)	101 (20%)	17 (17%)
$100,00–$149,000	290 (20%)	108 (22%)	20 (20%)
$150,000 and over	187 (13%)	74 (15%)	8 (7.9%)
Unknown	4	8	
Tenure at main job (years)	4.2 (2.1, 7.5)	3.8 (1.8, 7.2)	4.2 (2.2, 7.5)
Unknown	58	30	3

a *n* (%); median (IQR).

**TABLE 3:** Marginal discrimination parameters, long-form Thriving from Work Questionnaire.

Item	General Thriving form Work	Psychological and emotional well-being from work	Social well-being from work	Work-life integration	Basic needs for thriving	Experience of work and job design	Health and physical and mental well-being from work
My work adds meaning to my life (meaning)	1.42	1.36					
My job allows me to achieve my full potential (potential)	1.72	1.01					
I love my job (enthusiasm)	1.84	1.25					
The kind of work I do makes me happy (happiness)	1.73	1.42					
I am satisfied with my job (satisfaction)	1.84	1.15					
My work adds to my overall life satisfaction (life satisfaction)	1.79	1.25					
I feel supported by the people I work with (support)	2.34		1.03				
I feel valued by the people I work with (valued)	2.52		1.02				
I am treated fairly at work (fairness)	2.74		0.46				
I am treated with respect at work (respect)	2.92		0.49				
At work, I feel like belong (belonging)	2.66		0.31				
I receive recognition at work for my accomplishments (recognition)	2.36						
I can voice concerns or make suggestions at work without getting into trouble (voice)	2.39						
I can easily manage my job as well as attend to the needs of my family (work family)	1.51			1.44			
I feel safe getting to and from work (commute)	1.08			0.50			
I can achieve a healthy balance between my work and my life outside of work (work life)	1.59			1.23			
I am paid fairly for the job I do (pay)	1.52				0.86		
I am satisfied with the amount of paid leave I can take to care for myself or family members (benefits)	1.43				1.02		
I feel my job is secure (Job Security)	1.52				0.47		
I have good promotion opportunities (progression)	1.38				0.53		
I am happy with how much input I have in decisions that affect my work (autonomy)	2.31					0.36	
I have adequate control over the pace of my work (work intensity)	1.57					1.06	
I am happy with how much control I have over my work schedule (schedule)	1.68					0.71	
I can easily manage the demands of my job (demands)	1.60					0.67	
I have access to the resources I need to do my job well (resources)	2.06					0.35	
I feel physically safe at work (physical Safety)	1.14						1.67
I feel psychologically safe at work (psychological safety)	1.92						0.92
I feel excessive levels of stress from my work (stress)	−0.99						−0.45
I worry that I will get hurt at work (injury)	0.66						1.43
After I leave work, I have enough energy to do the things I want or need to do (energy)	1.47						0.21
ECV	71.2%	9.8%	3.3%	4%	3%	3%	5.7%
Empirical reliability	0.93	0.82	0.62	0.70	0.52	0.54	0.62

*Note:* Model fit: M2_(*df = 257*)_ = 854.9; RMSEA = 0.05 (90% CI-LB = 0.04); SRMSR = 0.05; CFI = 0.96.

Abbreviation: ECV, explained common variance.

**TABLE 4:** Short-form discrimination and intercepts.

	Discrimination parameter	Category intercepts				
		1	2	3	4	5
I love my job (enthusiasm)	1.73	3.56	2.07	0.49	−0.75	−2.53
I am treated fairly at work (fairness)	2.54	7.72	5.45	3.39	1.03	−1.80
I can achieve a healthy balance between my work and life outside of work (work-life)	1.70	6.08	3.40	1.61	−0.14	−2.17
I am paid fairly for the job I do (pay)	1.51	3.74	2.26	0.98	−0.37	−1.92
I am happy with how much input I have in decisions that affect my work (autonomy)	2.29	5.60	3.42	1.54	−0.42	−2.62
I can easily manage the demands of my job (demands)	1.73	6.2	4.75	2.59	0.32	−2.07
I can voice my concerns or make suggestions at work without getting into trouble (voice)	2.20	5.96	4.17	2.36	0.26	−1.84
I feel psychologically safe at work (psychological safety)	1.86	6.64	4.77	3.14	1.38	−0.51
Empirical reliability	0.87					

Model fit: M2_(*df* = 20)_ = 253.81; RMSEA = 0.09 (90% CI-LB = 0.08); SRMSR = 0.05; CFI = 0.97.

**TABLE 5:** Construct validity: Pearson's correlations.

	TFW (short-form)	TFW (long-form)
TFW (long-form)	0.97	
Gallup Cantrell's Ladder	0.36	0.37
Perception of best job	0.48	0.48
Life satisfaction	0.42	0.43
Mental health	−0.45	−0.45

Abbreviation: TFW, Thriving from Work.
