# Supplementary material for: Thriving from Work Questionnaire: Dimensionality, reliability, and validity of the long and short form questionnaires
Source: Am J Ind Med. Author manuscript; Available in PMC 2026 Apr 3. (PMC13048217; doi:10.1002/ajim.23465)
Supplement: Appendix 1 [file NIHMS2145150-supplement-Appendix_1.pdf]

Peters SE, Gundersen DA, Katz JN, Sorensen G, Wagner GR. Thriving from Work Questionnaire: dimensionality, reliability, and validity of the long and short form questionnaires. Am J Ind Med. 2023;66:281-296. doi:10.1002/ajim.23465

Appendix 1. Descriptive Statistics for Candidate Items (n=1479)

Item	Never n(%)	Rarely n(%)	Some-times n(%)	Usually n(%)	Almost Always n(%)	Always n(%)	Missing/ NA n
My work gives me a sense of purpose.	103 (7%)	164 (11%)	299 (20%)	288 (19%)	339 (23%)	284 (19%)	2
My work adds meaning to my life.	114 (7.7%)	212 (14%)	301 (20%)	300 (20%)	308 (21%)	236 (16%)	8
My work makes a meaningful contribution to society.	61 (4.1%)	147 (10%)	277 (19%)	346 (23%)	334 (23%)	311 (21%)	3
My job allows me to achieve my full potential.	127 (8.6%)	236 (16%)	300 (20%)	307 (21%)	309 (21%)	195 (13%)	5
My work allows me to develop new knowledge and skills.	42 (2.8%)	148 (10%)	329 (22%)	323 (22%)	344 (23%)	291 (20%)	2
I feel supported by the people I work with.	17 (1.2%)	60 (4.1%)	212 (15%)	369 (25%)	482 (33%)	316 (22%)	23
I feel valued by the people I work with.	15 (1.0%)	74 (5.1%)	226 (15%)	339 (23%)	474 (32%)	331 (23%)	20
My work is valued by others.	15 (1.0%)	73 (4.9%)	208 (14%)	381 (26%)	480 (33%)	318 (22%)	4
I am treated fairly at work.	11 (0.7%)	46 (3.1%)	142 (9.7%)	349 (24%)	520 (35%)	401 (27%)	10
I am treated with respect at work.	9 (0.6%)	29 (2.0%)	140 (9.5%)	325 (22%)	517 (35%)	448 (31%)	11
I am bullied, harassed, or humiliated at work.	1,096 (76%)	249 (17%)	60 (4.1%)	12 (0.8%)	17 (1.2%)	14 (1.0%)	31
I am comfortable being myself at work	31 (2.1%)	69 (4.7%)	174 (12%)	373 (25%)	435 (29%)	393 (27%)	4
At work, I feel like I belong.	41 (2.8%)	98 (6.7%)	262 (18%)	352 (24%)	414 (28%)	300 (20%)	12
At work, my opinions matter.	37 (2.5%)	99 (6.7%)	273 (19%)	367 (25%)	395 (27%)	301 (20%)	7
No one cares about my opinions at work.	571 (39%)	524 (36%)	198 (14%)	83 (5.7%)	47 (3.2%)	27 (1.9%)	29
I receive recognition at work for my accomplishments.	59 (4.0%)	167 (11%)	390 (27%)	343 (23%)	299 (20%)	204 (14%)	17
I can voice concerns or make suggestions at work without getting into trouble.	27 (1.8%)	73 (5.0%)	188 (13%)	392 (27%)	417 (29%)	366 (25%)	16
My work allows me to contribute to the happiness and wellbeing of others.	41 (2.8%)	126 (8.6%)	288 (20%)	332 (23%)	395 (27%)	281 (19%)	16
I feel supported by my coworkers.	14 (1.0%)	55 (3.8%)	188 (13%)	346 (24%)	483 (33%)	357 (25%)	36
I feel supported by my managers/supervisors.	20 (1.4%)	86 (6.0%)	217 (15%)	314 (22%)	448 (31%)	356 (25%)	38
I receive useful and timely feedback at work from my managers/supervisors.	35 (2.4%)	159 (11%)	268 (19%)	353 (24%)	363 (25%)	263 (18%)	38

I feel valued by my coworkers.	15 (1.0%)	47 (3.3%)	191 (13%)	333 (23%)	487 (34%)	369 (26%)	37
I feel valued by my managers/supervisors.	20 (1.4%)	97 (6.7%)	209 (14%)	315 (22%)	422 (29%)	380 (26%)	36
I feel valued by other people I interact with at work, such as customers, clients, students, patients (any other people who are NOT your supervisors or coworkers).	11 (0.8%)	63 (4.5%)	197 (14%)	405 (29%)	458 (32%)	276 (20%)	69
My work is valued by my coworkers.	16 (1.1%)	59 (4.1%)	187 (13%)	323 (22%)	496 (34%)	359 (25%)	39
My work is valued by my managers/supervisors.	14 (1.0%)	79 (5.5%)	178 (12%)	335 (23%)	453 (31%)	383 (27%)	37
My work is valued by other people I interact with work, such as customers, clients, students, patients (any other people who are NOT your supervisors or coworkers).	15 (1.1%)	50 (3.5%)	214 (15%)	374 (26%)	474 (33%)	291 (21%)	61
I am treated fairly by my coworkers.	10 (0.7%)	27 (1.9%)	110 (7.6%)	302 (21%)	543 (38%)	448 (31%)	39
I am treated fairly by my managers/supervisors.	13 (0.9%)	42 (2.9%)	166 (12%)	319 (22%)	480 (33%)	422 (29%)	37
I am treated fairly by people I interact with at work, such as customers, clients, students, patients (any other people who are NOT your supervisors or coworkers)	11 (0.8%)	40 (2.8%)	150 (11%)	398 (28%)	505 (36%)	308 (22%)	67
I am treated with respect by my coworkers.	7 (0.5%)	25 (1.7%)	115 (8.0%)	277 (19%)	541 (37%)	481 (33%)	33
I am treated with respect by my supervisors.	14 (1.0%)	35 (2.4%)	141 (9.8%)	269 (19%)	501 (35%)	481 (33%)	38
I am treated with respect by other people I interact with at work, such as customers, clients, students, patients (any other people who are NOT your supervisors or coworkers).	9 (0.6%)	37 (2.6%)	149 (11%)	401 (28%)	512 (36%)	304 (22%)	67
I can easily manage my job as well as attend to my needs and the needs of my family.	8 (0.5%)	63 (4.3%)	214 (15%)	436 (30%)	468 (32%)	281 (19%)	9
I can achieve a healthy balance between my work and my life outside of work.	13 (0.9%)	103 (7.0%)	251 (17%)	416 (28%)	426 (29%)	269 (18%)	1
My family and friends value the work I do.	40 (2.8%)	119 (8.2%)	283 (19%)	339 (23%)	364 (25%)	307 (21%)	27

I worry about things at work when I am not working.	186 (13%)	416 (28%)	509 (35%)	172 (12%)	131 (8.9%)	59 (4.0%)	6
Traveling to and from work is stressful for me.	395 (30%)	478 (37%)	238 (18%)	85 (6.6%)	55 (4.2%)	45 (3.5%)	183
Traveling to and from work is easy and stress-free.	31 (2.3%)	63 (4.8%)	204 (15%)	328 (25%)	362 (27%)	332 (25%)	159
I feel safe getting to and from work.	14 (1.1%)	23 (1.7%)	82 (6.2%)	255 (19%)	419 (32%)	531 (40%)	155
I am paid fairly for the job I do.	78 (5.3%)	160 (11%)	267 (18%)	352 (24%)	333 (23%)	283 (19%)	6
My pay meets my needs and the needs of my family.	67 (4.6%)	139 (9.5%)	252 (17%)	364 (25%)	335 (23%)	310 (21%)	12
I am not paid enough money to make ends meet.	527 (37%)	365 (25%)	264 (18%)	109 (7.6%)	89 (6.2%)	84 (5.8%)	41
I am satisfied with the employee benefits provided through my work, such as access to health insurance, life insurance, a pension or retirement saving plan.	104 (7.7%)	127 (9.4%)	177 (13%)	293 (22%)	334 (25%)	323 (24%)	121
I am satisfied with the amount of paid vacation days I get.	128 (9.6%)	125 (9.3%)	162 (12%)	261 (19%)	308 (23%)	356 (27%)	139
I am satisfied with the amount of paid leave I can take to care for myself or family members.	115 (8.5%)	136 (10%)	175 (13%)	290 (21%)	316 (23%)	318 (24%)	129
I feel my job is secure.	39 (2.6%)	76 (5.1%)	198 (13%)	367 (25%)	413 (28%)	384 (26%)	2
I have good opportunities for promotion.	152 (11%)	326 (23%)	296 (21%)	263 (19%)	206 (15%)	148 (11%)	88
I am grateful for my job.	15 (1.0%)	41 (2.8%)	169 (11%)	289 (20%)	369 (25%)	593 (40%)	3
I am happy with how much input I have in decisions that affect my work.	39 (2.7%)	135 (9.2%)	265 (18%)	392 (27%)	376 (26%)	262 (18%)	10
I can solve problems at work without having to ask for permission.	11 (0.7%)	36 (2.4%)	205 (14%)	400 (27%)	548 (37%)	275 (19%)	4
I have adequate control over the pace of my work.	25 (1.7%)	107 (7.2%)	214 (14%)	410 (28%)	434 (29%)	289 (20%)	
I have control over how my daily work is done.	23 (1.6%)	73 (4.9%)	202 (14%)	411 (28%)	458 (31%)	311 (21%)	1
I am happy with how much control I have over my work schedule.	41 (2.8%)	109 (7.5%)	227 (16%)	308 (21%)	419 (29%)	358 (24%)	17
I can schedule a day off or take vacation when I want or need to.	30 (2.0%)	106 (7.2%)	215 (15%)	335 (23%)	416 (28%)	364 (25%)	13
I can take unpaid leave if I need to.	79 (5.7%)	160 (11%)	241 (17%)	294 (21%)	278 (20%)	342 (25%)	85

I can easily manage the demands of my job.	12 (0.8%)	28 (1.9%)	164 (11%)	452 (31%)	533 (36%)	290 (20%)	
I have enough time, within my normal working hours, to get my job done.	21 (1.4%)	80 (5.4%)	221 (15%)	388 (26%)	486 (33%)	280 (19%)	3
I have more work to do than I can complete during paid work hours.	178 (12%)	415 (28%)	443 (30%)	178 (12%)	155 (11%)	92 (6.3%)	18
I have access to the resources I need to do my job well.	14 (0.9%)	49 (3.3%)	166 (11%)	375 (25%)	500 (34%)	375 (25%)	
I know enough about what is going on in my company/ organization to do my job well.	9 (0.6%)	51 (3.5%)	168 (11%)	399 (27%)	510 (35%)	335 (23%)	7
I have the skills and knowledge I need to do my job well.	9 (0.6%)	16 (1.1%)	86 (5.8%)	271 (18%)	600 (41%)	496 (34%)	1
My physical work environment (work space, light, temperature) is set up in a way that helps me do my job well.	21 (1.4%)	76 (5.2%)	204 (14%)	376 (26%)	435 (30%)	349 (24%)	18
Noise at work interferes with my ability to get the job done.	385 (27%)	569 (40%)	295 (21%)	96 (6.7%)	62 (4.3%)	30 (2.1%)	42
I feel physically safe at work.	5 (0.3%)	19 (1.3%)	70 (4.8%)	174 (12%)	393 (27%)	795 (55%)	23
I feel psychologically safe at work.	10 (0.7%)	35 (2.4%)	107 (7.3%)	267 (18%)	432 (29%)	615 (42%)	13
I feel excessive levels of stress from my work.	189 (13%)	466 (32%)	523 (36%)	149 (10%)	94 (6.4%)	50 (3.4%)	8
I worry that I will get hurt at work.	819 (57%)	403 (28%)	137 (9.6%)	37 (2.6%)	19 (1.3%)	19 (1.3%)	45
After I leave work, I have enough energy to do the things I want or need to do.	44 (3.0%)	158 (11%)	379 (26%)	457 (31%)	322 (22%)	109 (7.4%)	10
My work contributes in a positive way to my wellbeing.	56 (3.8%)	179 (12%)	353 (24%)	386 (26%)	307 (21%)	192 (13%)	6
I find work emotionally exhausting.	162 (11%)	440 (30%)	505 (34%)	183 (12%)	120 (8.2%)	62 (4.2%)	7
I find work physically exhausting.	360 (25%)	457 (31%)	384 (26%)	152 (10%)	69 (4.7%)	40 (2.7%)	17
I am too tired after work to enjoy things.	161 (11%)	494 (34%)	563 (38%)	135 (9.2%)	81 (5.5%)	38 (2.6%)	7
I feel engaged by my work.	50 (3.4%)	115 (7.8%)	329 (22%)	371 (25%)	365 (25%)	247 (17%)	2
At work, my mind is focused on my job.	22 (1.5%)	66 (4.5%)	203 (14%)	503 (34%)	429 (29%)	255 (17%)	1
I am enthusiastic about my work.	65 (4.4%)	181 (12%)	319 (22%)	376 (25%)	342 (23%)	195 (13%)	1
I love my job.	108 (7.3%)	189 (13%)	341 (23%)	302 (20%)	320 (22%)	215 (15%)	4

My job is pointless (has no useful purpose).	712 (49%)	375 (26%)	186 (13%)	87 (6.0%)	61 (4.2%)	28 (1.9%)	30
My job is boring.	264 (18%)	434 (30%)	436 (30%)	172 (12%)	101 (6.9%)	59 (4.0%)	13
The kind of work I do makes me happy.	72 (4.9%)	169 (11%)	335 (23%)	337 (23%)	337 (23%)	227 (15%)	2
My job makes me happy.	76 (5.1%)	189 (13%)	351 (24%)	373 (25%)	292 (20%)	195 (13%)	3
I am satisfied with my job.	58 (3.9%)	122 (8.3%)	295 (20%)	361 (24%)	400 (27%)	240 (16%)	3
I am satisfied with the kind of work I do.	56 (3.8%)	125 (8.5%)	291 (20%)	346 (23%)	401 (27%)	260 (18%)	
My work adds to my overall life satisfaction.	82 (5.6%)	182 (12%)	335 (23%)	346 (23%)	318 (22%)	212 (14%)	4
At work, I have the opportunity to do what I do best every day.	85 (5.8%)	124 (8.4%)	312 (21%)	374 (25%)	357 (24%)	224 (15%)	3
