# Supplementary material for: Thriving from Work Questionnaire: Dimensionality, reliability, and validity of the long and short form questionnaires
Source: Am J Ind Med. Author manuscript; Available in PMC 2026 Apr 3. (PMC13048217; doi:10.1002/ajim.23465)
Supplement: Appendix 2 [file NIHMS2145150-supplement-Appendix_2.pdf]

Peters SE, Gundersen DA, Katz JN, Sorensen G, Wagner GR. Thriving from Work Questionnaire: dimensionality, reliability, and validity of the long and short form questionnaires. Am J Ind Med. 2023;66:281-296. doi:10.1002/ajim.23465

Appendix 2. Long- and Short- Form Thriving from Work Questionnaires

Thriving from Work Questionnaire – Long-form

The following items relate to how you perceive the work you do day-to-day.
If you have more than one job, please consider your current job that is most IMPORTANT to you when responding. Please think about this same job when you are answering all of the questions.

Indicate how often, if at all, you have generally felt that way about your work over the last month.

Please read the questions carefully and give your best honest answer.

Select one response for each item.

Response categories: Never, Rarely, Sometimes, Usually, Almost Always, Always

Domain	Item
Work-related Emotional & Psychological Well-being	1. I love my job.<br>2. My work adds meaning to my life.<br>3. My job allows me to achieve my full potential.<br>4. The kind of work I do makes me happy.<br>5. I am satisfied with my job.<br>6. My work adds to my overall life satisfaction.
Social Well-being from Work	7. I am treated fairly at work.<br>8. I feel supported by the people I work with.<br>9. I feel valued by the people I work with.<br>10. I am treated with respect at work.<br>11. At work, I feel like I belong.
Work-life integration	12. I can achieve a healthy balance between my work and my life outside of work.<br>13. I can easily manage my job as well as attend to my needs and the needs of my family.<br>14. I feel safe getting to and from work.
Basic Needs for Thriving	15. I am paid fairly for the job I do.<br>16. I am satisfied with the amount of paid leave I can take to care for myself or family members.<br>17. I feel my job is secure.<br>18. I have good opportunities for promotion.
Job design & experience of work	19. I am happy with how much input I have in decisions that affect my work.<br>20. I can easily manage the demands of my job.<br>21. I have adequate control over the pace of my work.<br>22. I am happy with how much control I have over my work schedule.<br>23. I have access to the resources I need to do my job well.
Health & physical and mental well-being from work	24. I feel psychologically safe at work.<br>25. I feel physically safe at work.<br>26. I feel excessive levels of stress from my work.*<br>27. After I leave work, I have enough energy to do the things I want or need to do.<br>28. I worry that I will get hurt at work.*
	29. I can voice concerns or make suggestions at work without getting into trouble.<br>30. I receive recognition at work for my accomplishments.

*Reverse Code

For an electronic survey, we would suggest random ordering of the questions within the domains, and random ordering of the domains.

Peters SE, Gundersen DA, Katz JN, Sorensen G, Wagner GR. Thriving from Work Questionnaire: dimensionality, reliability, and validity of the long and short form questionnaires. Am J Ind Med. 2023;66:281-296. doi:10.1002/ajim.23465

Thriving from Work Questionnaire – Short-form

The following items relate to how you perceive the work you do day-to-day.
If you have more than one job, please consider your current job that is most IMPORTANT to you when responding. Please think about this same job when you are answering all of the questions.

Indicate how often, if at all, you have generally felt that way about your work over the last month.

Please read the questions carefully and give your best honest answer.

Select one response for each item.

1. I love my job.
2. I am treated fairly at work.
3. I can achieve a healthy balance between my work and my life outside of work.
4. I am paid fairly for the job I do.
5. I am happy with how much input I have in decisions that affect my work.
6. I can easily manage the demands of my job.
7. I feel psychologically safe at work.**
8. I can voice concerns or make suggestions at work without getting into trouble.

Response categories: Never, Rarely, Sometimes, Usually, Almost Always, Always

** Depending on the nature of the industry or scope of research/practice, this item can be replaced with "*I feel physically safe at work*."
